# Application of Biophysical Techniques to Investigate the Interaction of Antimicrobial Peptides With Bacterial Cells

**DOI:** 10.3389/fmedt.2020.606079

**Published:** 2020-12-15

**Authors:** Maria Luisa Gelmi, Luca Domenico D'Andrea, Alessandra Romanelli

**Affiliations:** ^1^Department of Pharmaceutical Sciences, University of Milan, Milan, Italy; ^2^Institute of Biostructures and Bioimaging, CNR, Turin, Italy

**Keywords:** peptide, antimicrobial, bacteria, biophysical techniques, mechanism of action

## Abstract

Gaining new understanding on the mechanism of action of antimicrobial peptides is the basis for the design of new and more efficient antibiotics. To this aim, it is important to detect modifications occurring to both the peptide and the bacterial cell upon interaction; this will help to understand the peptide structural requirement, if any, at the base of the interaction as well as the pathways triggered by peptides ending in cell death. A limited number of papers have described the interaction of peptides with bacterial cells, although most of the studies published so far have been focused on model membrane-peptides interactions. Investigations carried out with bacterial cells highlighted the limitations connected to the use of oversimplified model membranes and, more importantly, helped to identify molecular targets of antimicrobial peptides and changes occurring to the bacterial membrane. In this review, details on the mechanism of action of antimicrobial peptides, as determined by the application of spectroscopic techniques, as well as scattering, microscopy, and calorimetry techniques, to complex systems such as peptide/bacteria mixtures are discussed.

## Introduction

Antimicrobial peptides (AMPs) are produced by all organisms and represent the first line of defense against attack by external pathogens (1). They are expressed or may be produced upon encountering a stimulus; interestingly, each organism produces its own set of peptides, tailored for the “enemy” that has to be killed. A feature that is common to most antimicrobial peptides is the presence of positively charged amino acids, which are essential for establishing an interaction with the negatively charged cell wall of bacteria and fungi. The distribution of charged and hydrophobic residues within the peptide sequence is thought to be functional to the interaction of peptides with bacteria and to their activity. In this review we focus on biophysical studies aimed at exploring the interaction of peptides with bacterial cells. It is our opinion that, although more complicated to set up, these studies will result in a more reliable picture of the mechanism of action of AMPs as compared to studies with systems that mimic cell membranes. Model systems are mainly composed of lipid mixtures, whereas bacterial cells contain a variety of molecules, including those other than lipids, sugars, and proteins. In principle, interactors of antimicrobial peptides could be found among all these molecules; in addition, the behavior of any of these molecules within the bacterial membrane may be different as compared to that of the same isolated molecules. The different chemical composition of membranes also affects their fluidity, which is crucial to different biological processes and is tuned by cells depending on external conditions. Results obtained with model membranes are strictly dependent on the set up of the experiment; it is demonstrated that the composition of the lipid mixture affects the structure of peptides, their dynamic, and their orientation with respect to membranes (2). For example, the peptide maculatin 1.1 is reported to insert into neutral phosphatidylcholine membranes assuming a helical conformation, whereas it remains locked on the surface of bilayers when anionic lipids are used. The helical content is dependent on the lipid composition (3).

Challenges imposed by the use of live bacterial cells derive from the need to adapt biophysical techniques to heterogeneous and evolving samples; ideally, measurements should be carried out on live cells, therefore experimental conditions compatible with measurements and cell life need to be sought out (4). In many cases, high background noise due to the presence of salts or micrometric cells may be an issue. Another critical issue is that not all bacteria may be managed in all laboratories, which limits the studies that can be performed in a standard chemistry lab. Biosafety levels of bacteria need to be carefully checked; in general, bacteria that present minimal potential hazards for the lab user and the environment (biosafety level 1, as certified by ATCC), such as non-pathogenic *E. coli* strains, may be managed with proper care in most laboratories. In the last few years, an increasing number of publications have appeared and details on the mechanism of action of peptides are being discovered with the support of biophysical techniques.

**Figure 1:**
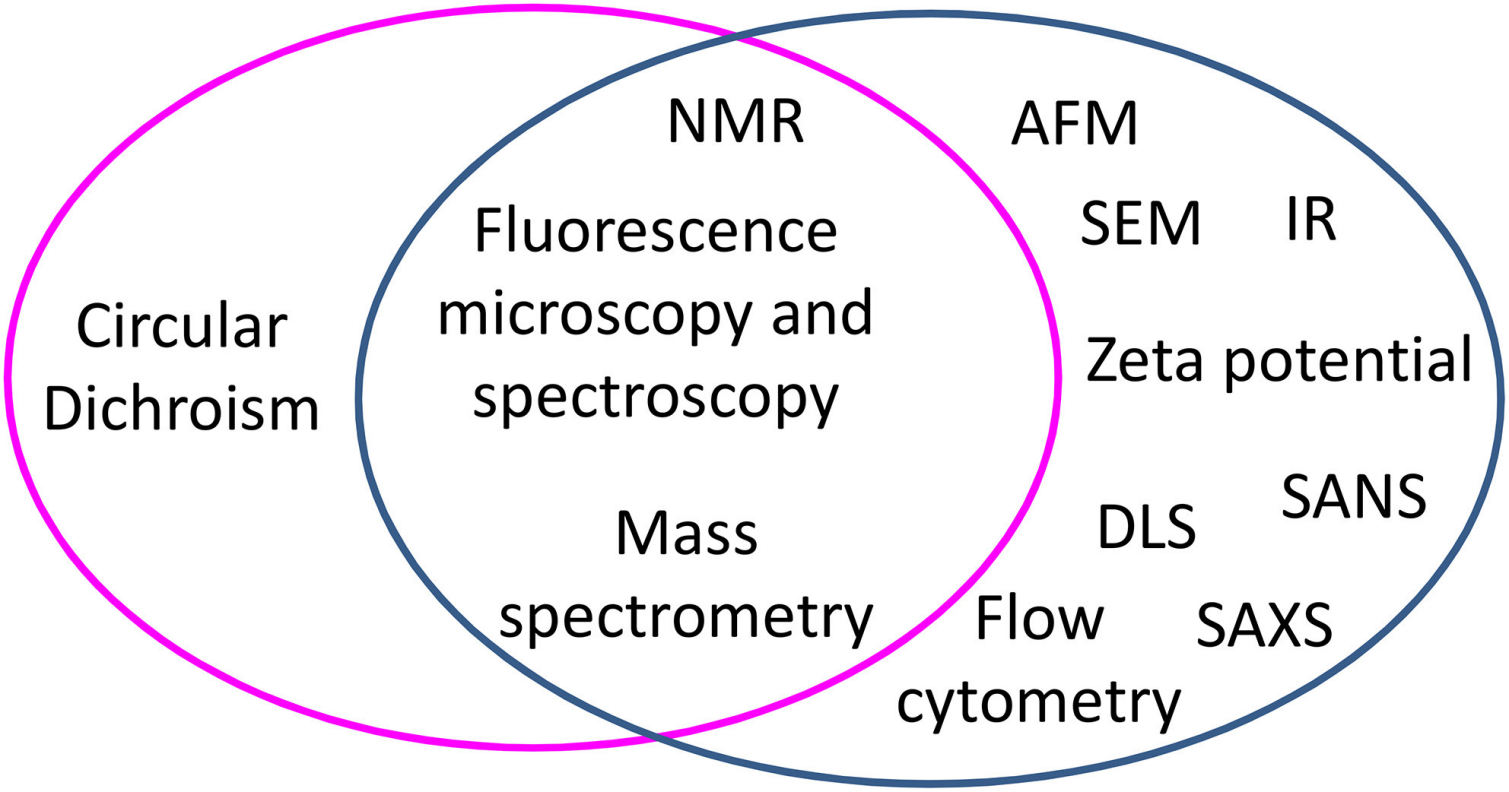
Biophysical techniques applied to study the mechanism of action of peptides in experiments performed with bacterial cells, grouped by techniques used to monitor changes occurring to peptides (magenta circle) and techniques used to monitor changes at bacterial cells (blue circle). At the cross, techniques useful to both aims are listed.

Mechanism studies can be carried out starting from two different perspectives: either by looking at changes occurring to peptides or by looking at changes occurring to cells. Figure 1 groups biophysical techniques in three main families: one including techniques exploited to monitor changes occurring at peptides in the presence of bacterial cells, one including techniques used to detect changes occurring at bacterial cells in the presence of AMPs, and one including techniques useful for both scopes. Understanding the changes occurring to the peptides upon contact with bacterial cells yields information at the molecular level on the factors that determine the initial interaction and trigger cell death. On the other hand, studies carried out looking at cells yield information on the effect of the AMPs on features such as the morphology of the cell; in many cases, these changes can be related to molecular events triggered by peptides. For example, the appearance of filaments in bacterial cells treated with AMPs is associated to the inhibition of cell division, that in turn depends on the interaction of AMPs with specific targets (5–7). Some techniques are suitable to investigate changes occurring both to peptides and cells; as an example, Nuclear Magnetic Resonance (NMR), that looks at changes in the chemical shift of magnetically active nuclei such as ^1^H, ^13^C, and ^31^P, is exploited to determine the structure of peptides that interact with cells, but is also used to detect the binding of peptides to cells (8–11). Similarly, fluorescence microscopy can be used to follow labeled peptides inside cells or to monitor the permeabilization of bacterial cells expressing fluorescent proteins able to cross damaged membranes (12–18). Other techniques, such as scattering techniques (DLS, SANS, SAXS) or AFM or TEM, are employed to monitor only changes at bacterial cells (19, 20). In this review, we describe the application of selected biophysical techniques to the study of the mechanism of action of AMPs.

## Structural Studies of Peptides Interacting with Bacterial Cells

Structural changes of peptides may be investigated by different techniques, such as **Circular Dichroism** (CD) that affords information on the secondary structure of peptides, or **NMR** that gives information on their three-dimensional structure. Most of the studies are reported for peptides that interact with *E. coli* cells, some studies are reported for peptides interacting with fungi and, to the best of our knowledge, only one paper reports the structure of peptides in the presence of a Gram-positive bacterium such as *S. epidermidis* (21–24). CD studies were initially reported on magainin 2 and cecropin A in the presence of *E. coli* cells, revealing that these peptides do not show a preferred conformation in buffer, but rapidly fold into α helices upon interaction with cells (21). A comparison with data obtained for the same peptides in the presence of *E. coli* lipopolysaccharide (LPS) reveals similar secondary structures, driving to the conclusion that the folding process depends to a large extent on the interaction of peptides with LPS, that is also the main component of the Gram-negative bacterial outer membrane. Similar behavior was observed for temporin L, TBKKG6A, and LG-21 (11, 22, 23). Interestingly, the peptide temporin B that is inactive against *E. coli* folds with purified LPS but not with cells (11). The peptide PG-1 is reported to fold into a beta hairpin upon interaction with *E. coli* cells (24).

CD studies to detect structural changes of peptides incubated with the Gram-positive *S. epidermidis* cells are reported for TBKKG6A, temporin B, and temporin L; in the presence of cells the first two peptides remain unfolded, while temporin L shows weak signals, suggesting the presence of a helical structure (22). Electrostatic interactions seem to drive peptide folding, being crucial for establishing contact with Gram-negative bacterial cells; in the case of Gram-positive bacteria, whose outer membrane shows a lower negative charge, hydrophobic interactions seem to play a more important role (25). CD studies have also been carried out with fungi: peptides MAP and cecropin B are reported to fold into helices in the presence of *C. albicans*; the protein NFAP from *N. fischeri* or its synthetic derivative spanning NFAP γ core, which possesses a very stable β pleated structure stabilized by disulfide bonds, do not change their conformation when incubated with *C. herbarium* (26, 27).

More detailed information on the structure of peptides has been derived from NMR studies. The three-dimensional structure of the peptide TBKKG6A determined by NMR either with *E. coli* LPS or with *E. coli* cells reveals that the peptide appears as a straight helix in the presence of LPS, while it is a kinked helix when incubated with cells (11, 28). These data support the idea that different components of the bacterial membrane concur to stabilize the interaction of AMPs with bacterial cells. **Rotational-echo double-resonance (REDOR) NMR** was applied to characterize complexes formed between ^19^F labeled vancomycin-like peptides and *S. aureus* cells labeled at the peptidoglycan (PG) with ^15^N and ^13^C (10). The ^13^C-^19^F and ^15^N-^19^F distances from the REDOR experiments were used to build model structures for all glycopeptide-PG complexes, allowing for the dissection of the vancomycin in five distinct fragments and assignment to each of a role in the interaction with PG.

## Binding Studies

The binding of peptides to cells can be investigated by NMR, fluorescence, zeta potential measurements, and Isothermal Titration Calorimetry (ITC). Through NMR, changes occurring at membranes in live bacterial cells upon AMP binding can be detected. **Solid state** ^**31**^**P NMR** is used to examine the interaction of peptides with the phospholipids of live bacteria. As the number of phosphate groups in a bacterial membrane is estimated to overwhelm that found in bacterial RNA, ^31^P is a suitable probe of membrane dynamics. The first paper on this topic dates back to 2000 and reports on the interactions of caerin 1.1, caerin 4.1, and maculatin 1.1 with the Gram-positive bacteria *B. cereus* and *S. epidermidis*. Caerin 4.1, which is not active against *B. cereus* and *S. epidermidis*, does not affect the ^31^P spectra. Caerin 1.1 and maculatin 1.1 instead induce isotropic peak formation, due to membrane disruption (9). More recently, ^**2**^**H solid state NMR** has been exploited to detect the interaction of the peptide MSI-78 with *E. coli* cells that incorporated high levels of ^2^H labels specifically into membranes (8). These special cells, obtained by genetic manipulation, do not synthesize or metabolize fatty acids and are able to incorporate ^2^H labeled palmitic acid from the growth media and produce ^2^H labeled saturated phospholipids. Lipid bilayer disruption is followed by the analysis of spectral moments; interestingly this phenomenon is observed in NMR at a peptide concentration well below that needed to inhibit cell growth. Furthermore, the peptide/lipid ratio required to induce lipid disordering in bacterial cells is much higher as compared to that needed to obtain the same effect in model lipid systems. This effect is likely due to the interaction of the peptide with other components of the cell envelope, such as LPS. Solid state ^2^H NMR studies are also reported to detect the interaction of caerin 1.1 and aurein 1.2 with ^2^H labeled *E. coli* and *B. subtilis* cells. In this case, membrane disordering effects were observed at MIC and were in agreement with data collected on model membranes.

**Fluorescence** spectroscopy using labeled peptides is used to determine the binding stoichiometry and binding constant of peptides to cells. Studies reported for TBKKG6A labeled with nitrobenzodiazole show that this peptide binds to *E. coli* cells in a 1 × 10^6^:1 (peptide:cell) stoichiometry; similar values are reported in the literature for other peptides (29–31). The binding constant of TBKKG6A to cells, expressed as a dissociation constant, reveals a very tight binding. Interestingly, this constant results to be higher as compared to that measured when *E. coli* LPS was used instead of cells. Experiments performed on the peptide temporin B, which is not active against *E. coli* cells, reveal no binding to cells but to LPS. Altogether, fluorescence data indicate that active peptides cover the entire bacterial surface; this hypothesis is sustained by data collected using techniques that are instead focused on detecting changes occurring to cells such as **Zeta potential** measurements. These measurements are used to determine the net surface charge of bacteria, which is a function of the media in which it is observed, depending on the concentration of molecules able to establish electrostatic interactions with cells. An increase in the zeta potential of *E. coli* cells incubated with antimicrobial peptides was observed, consistent with the idea that the peptides overlay the bacterial outer membrane. Neutralization may occur at different concentrations, as demonstrated for peptides BP100, pepR, and crotalicidin with *E. coli* cells (32, 33). BP100 neutralization occurs at MIC, while for pepR and crotalicidin (Ctn) zero-potential precedes the MIC; this different behavior suggests that in the case of pepR and Ctn cell death is triggered by factors other than membrane neutralization.

In addition, the binding of peptides to bacterial cells may be followed by IR, monitoring the fluidity of lipopolysaccharides and phospholipids within the membrane. In the **IR**, the 2851–2853 cm^−1^ band that is characteristic of the acyl chain order moves to lower wavelengths upon binding of peptides; gel-to-liquid phase transition temperatures also show minor changes (34, 35).

Thermodynamic investigations of the peptide-cell binding are also performed by **isothermal titration calorimetry (ITC)** (34). ITC experiments performed on the peptide Pep19-2.5 with Gram-negative and Gram-positive bacteria reveal that the binding process is exothermic; the amount of released heat is higher for Gram-negative than for Gram positive bacteria. Electrostatic interactions between the negative charges of LPS phosphates and positive charges of peptides lead to a strong exothermic reaction. In the case of Gram-positive bacteria, the cell wall contains teichoic acids, lipoteichoic acids and lipoproteins; the latest have phosphates in the form of diesters, shielded by the cell wall and therefore less accessible to peptides. This results in a lower energy release.

## Detection of Effects Occurring at the Membrane/Cell Surface

Fluorescence microscopy is widely used to follow peptides inside cells and visualize bacterial membranes permeabilization. Using *E. coli* cells expressing cytoplasmic GFP, it is possible to follow outer membrane (OM) permeabilization by the GFP signal decay; increase of the signal of the DNA stain Sytox green or Sytox red is related to cytoplasmic membrane (CM) permeabilization (13, 14). Using single cell fluorescence microscopy, it has been demonstrated in *E. coli* that mutations to the core oligosaccharide affect OM and CM permeabilization by cecropin A (12). In a different study, OM permeabilization has been detected by the profluorophore JF646, a small molecule (702 Da) emitting weak fluorescence in aqueous solution. When the AMP permeabilizes the outer membrane, JF646 enters the cytoplasm and covalently binds to the HaloTag protein emitting red fluorescence; permeabilization onset is detected with a 3s resolution (15, 16). When peptides are labeled, information on the localization and distribution of peptides may be obtained (17). Recently, laser scanning **Stimulated Emission Depletion** (STED) fluorescence microscopy was employed to detect the localization of BDP-FL labeled thanatin in *E. coli* cells: localization of the peptide in islands at the poles and across the cell was related to the interaction of the peptide with outer membrane proteins, a hypothesis confirmed by photoaffinity labeling experiments and *in vivo* by Bacterial Adenylate Cyclase Two-Hybrid (BACTH) system (18, 36). Using time-lapse **Fluorescence Lifetime Imaging Microscopy** (FLIM) on a fluorescently tagged melittin analog, transient disruption of the *E. coli* membrane was observed (37). Interestingly, the same experiment performed on synthetic membranes suggested the formation of stable pores.

By phase-contrast and time lapse microscopy, using fluorescent LL-37, the distribution, translocation, and retention of the AMP in *E. coli* cells was investigated (38). Snoussi observed that inhibition of bacterial growth is followed by a rapid translocation of peptides into cells. Experiments carried out at different peptide concentrations (above MIC and sub-MIC) reveal the relationship between the mean concentration of free peptides and the mean concentration of growing bacteria (inoculum effect); this relationship is also described by a mathematical model.

Permeabilization is also investigated by time-resolved **Flow Cytometry Assay** (FCA). Freire et al. demonstrated that pepR binds in a cooperative fashion and faster to the *E. coli* 25922 strain than to the *E. coli* K-12 strain, which presents a truncated LPS, while permeabilization takes place at a higher rate in *E. coli* K-12 than in *E. coli* 25922 (39). These studies highlight the role of the LPS composition in permeabilization kinetic by AMPs. Using labeled peptides, it is possible to correlate permeabilization and internalization kinetics. Time-resolved FCAs performed on Rhodamine B labeled Ctn and Ctn (15–34) show that binding and internalization processes occur until an equilibrium is reached; Ctn(15-34) uptake is faster and precedes bacterial membrane damage, suggesting that permeabilization occurs only upon achievement of a threshold surface concentration (33). In line with this, single cell images show that both peptides are mostly localized on the *E. coli* surface, but a small percentage co-localizes with the dye SYTOX green, suggesting a partial internalization.

Changes occurring to the bacterial cells surface are detected by microscopy (AFM; TEM, SEM). **Atomic Force Microscopy** (AFM) is frequently used to detect surface roughness and cell height; when measurements are carried out on dry samples, it is quite difficult to separate the effect due to drying from those caused by the AMPs. In some applications, images are taken on liquid samples. In a study on the peptide CM15, bacteria are imaged in aqueous solution; using high-speed atomic force, microscopy dynamic changes at a single cell level are recorded at a nanometric resolution (19). Following the average surface corrugation with the time, the authors proposed that bacterial death mediated by CM15 occurs in two stages: an incubation time, followed by an execution phase in which 50% of the damage occurring to cell happens. Mularski reports that melittin affects *K. pneumoniae* turgor pressure and cell wall elasticity, while it does not alter bacterial capsule thickness and organization, leading to the hypothesis that the capsule does not offer protection to *K. pneumoniae* against antimicrobial peptides (20).

**Figure 2:**
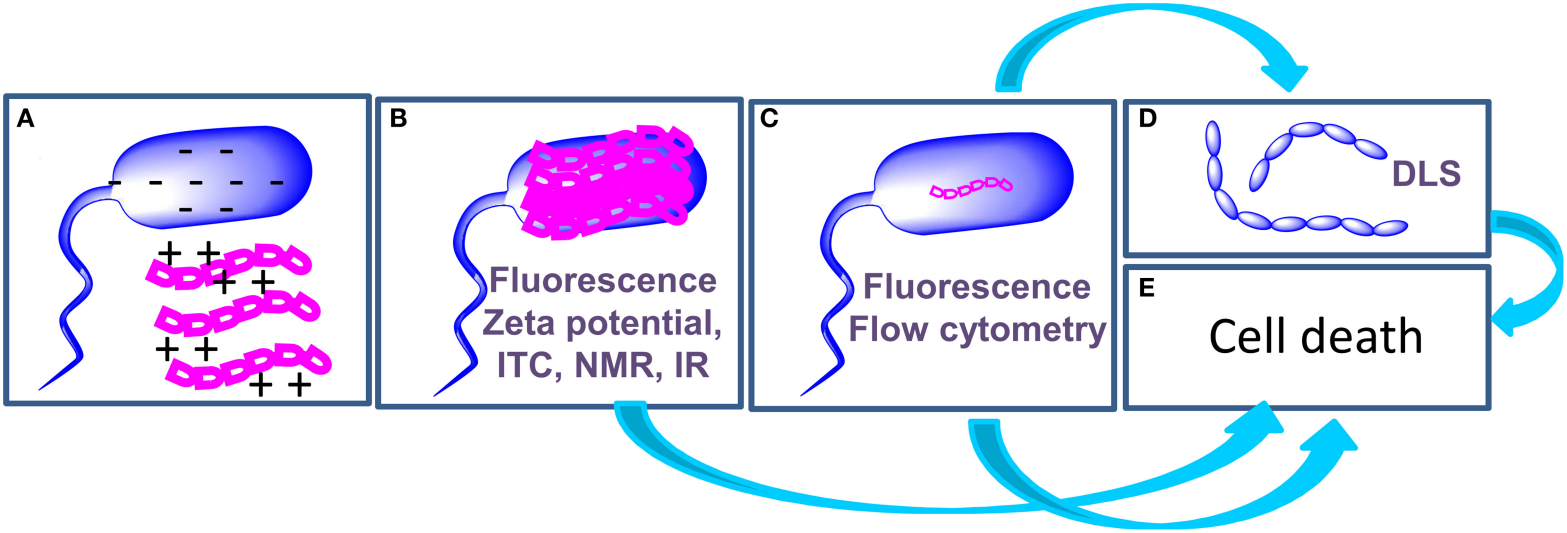
Schematic representation of the mechanism of action of AMPs and of the techniques (highlighted in purple in each panel) employed to investigate each step. AMPs (magenta) interact with bacterial cells (blue) through electrostatic interactions **(A)**. AMPs cover the surface of bacterial cells **(B)**; in some cases, this step triggers cell death **(E)**, in other cases AMPs enter the cell **(C)** to hit their targets and this step determines cell death **(E)**. When AMPs target proteins involved in cell division, agglutination occurs **(D)**.

Light scattering measurements are employed to detect aggregation of bacteria following treatment with AMPs (Figure 2D): Di Somma et al. report the formation of *E. coli* elongated structures with dimensions over 6000 nm upon incubation with temporin L (TL) (7). In the same work, changes to the structure of bacterial cells occurring at a nanoscale level, in the range of 2 to 300 nm, were observed by **Small Angle Neutron Scattering** measurements that disclose a change in the spatial arrangement of a protein involved in the interaction with TL.

## Target Identification

Identification of AMP targets in bacterial cells is the most challenging task in mechanism studies and usually requires integration of data from different experiments. While in many cases AMPs act by disrupting the bacterial membrane (33, 40), in other cases intracellular targets, such as DNA, RNA, and proteins, have been identified. Mass spectrometry is one of the most useful techniques to achieve this goal. As an example of application of mass spectrometry to the detection of protein targets in living bacterial cells, we here report on *in vivo* photolabelling. These experiments are based on the use of peptides containing a photoprobe (a L-4,4-diazarinylproline in place of one proline) and a biotin tag; these peptides are incubated with bacterial cells. Upon irradiation, the photoprobe captures the protein target of the AMP. Photolabelled proteins are separated by gel electrophoresis, digested in gel, and identified by tandem mass spectrometry. Identification of proteins involved in the biogenesis of the bacterial outer membrane as the interactors of antimicrobial peptides, such as L27-11 and thanatin, was achieved in this way (18, 41, 42).

## Conclusions

The application of biophysical techniques to study the interactions of AMPs with bacterial cells enabled the highlighting of some steps that are common to the mechanism of action of many peptides (Figure 2): (a) the initial interaction of AMPs with bacterial cells is mediated by electrostatic forces between peptides and the outer leaflet of bacteria. These interactions may result in a change in the structure of the peptide; (b) AMPs cover the outer surface of bacteria; (c) Internalization occurs with mechanisms and kinetics that depend on the composition of the bacterial membrane. Intracellular targets of AMPs are various, including proteins involved in cell division or in the synthesis/transport of LPS. (d) AMPs may cause cell agglutination; (e) AMPs may also cause cell death depending on the target. In order to draw conclusions on the mechanism of action of AMPs, the integration of data deriving from biophysical experiments with results of microbiological and genetic tests is of fundamental importance. We are confident that results of these studies will contribute to the exploitation of AMPs as drugs and to the design of new biomedical devices (43–46).

## Author Contributions

AR contributed to the conception of the manuscript. AR, LD'A, and MG contributed to the preparation and editing of the manuscript and approved the submitted version. All authors contributed to the article and approved the submitted version.

## Conflict of Interest

The authors declare that the research was conducted in the absence of any commercial or financial relationships that could be construed as a potential conflict of interest.
